# Sexual behaviour among casual workers in an international nightlife resort: a case control study

**DOI:** 10.1186/1471-2458-6-39

**Published:** 2006-02-21

**Authors:** Karen Hughes, Mark A Bellis

**Affiliations:** 1 Centre for Public Health, Faculty of Health and Applied Social Sciences, Liverpool John Moores University, Castle House, North Street, Liverpool, L3 2AY, UK

## Abstract

**Background:**

Young holidaymakers report increased sexual risk-taking abroad, yet little is currently known about the sexual behaviour of those who extend time abroad through casual work.

**Methods:**

Information on sexual behaviour was collected via an anonymous questionnaire administered to British bar and nightclub workers in Ibiza (cases, n = 92) and British people visiting Ibiza for holiday purposes only (controls, n = 868).

**Results:**

Four in five (80.5%) cases who arrived in Ibiza without a partner had sex during their stay and of these two thirds (65.5%) had unprotected sex. Cases were more likely to report sexual risk-taking in Ibiza than controls and reported greater numbers of sexual partners prior to their visit. However, they had fewer sexual partners per week of stay.

**Conclusion:**

Casual workers in bars and nightclubs abroad are a key risk group for sexual health and a potential conduit for the international spread of sexually transmitted infections. While they are an important target group for sexual health promotion, appropriately trained they are also ideally placed to deliver sexual health interventions to other young travellers.

## Background

Many countries are currently experiencing declining sexual health amongst young populations. In the UK, diagnoses of Chlamydia in 16–24 year olds have tripled since 1995[1], with increases also seen in a range of other countries (e.g. USA[2], Switzerland, Sweden[3]). The advent of widely affordable international travel means sex abroad is an increasingly important consideration in the spread of sexually transmitted infections[4,5]. Thus, time holidaying or working abroad may result in young people having sex with other travellers of their own nationality, with nationals of the country they are visiting or with individuals from other countries visiting the same destination. In fact, seasonal trends in sexual health show peaks in attendance at sexual health clinics and terminations of pregnancies following holiday periods[6,7]. Such peaks correspond to increased sexual activity and sexual risk taking among young people during summer holidays, often abroad and frequently in the context of high alcohol and drug use[8].

Typically, for the majority of young people summer breaks abroad are limited to one or two weeks. However many young people extend their holidays abroad by working in international resorts often for months at a time. While British casual staff in domestic holiday resorts report increased sexual activity whilst employed in seasonal work[9], no study has reported on sexual behaviour of individuals undertaking casual work abroad. However, such individuals are exposed to a recreational atmosphere for longer periods of time than their holidaymaking counterparts and are more prone to risk behaviours such as drug use[10]. Ibiza (Spain) is one of the most popular holiday resorts for young British people and an island with a long association with international nightlife. Consequently, here we examine the sexual behaviour of young British people working in bars and nightclubs in Ibiza and compare it with that of other UK residents travelling to the same destination for recreational purposes only.

## Methods

Cases were young British people (inclusion criteria, age 16–35; although the actual sample identified no under 18s) working in bars and nightclubs in Ibiza (n = 92; compliance 97.9%). Participants were recruited opportunistically by trained individuals familiar with the Ibiza nightlife environment visiting a random mix of bars and nightclubs in San Antonio town at the end of the 2002 summer season (September). Bar and nightclub staff were approached either while at work or during staff meetings, and at the point of first contact were asked if they had time to complete a short questionnaire. Those that did were provided with an explanation of the nature of the research, assured of their anonymity and survey confidentiality and asked if they would like to participate. Informed consent was obtained orally, with participants' rights to abstain from participation or withdraw consent at any time explained, and consent was documented along with compliance rates. Those that volunteered to participate were handed a questionnaire, pen and envelope and allowed to self-complete the questionnaire. Questionnaires were returned to researchers in sealed envelopes to preserve anonymity. No identifiable information was collected and research methods complied with the Helsinki Declaration.

Controls were young British people (inclusion criteria, age 16–35) visiting Ibiza for holiday purposes only during the same summer season and were recruited at Ibiza airport prior to their return to the UK (n = 868; compliance 92.6%)[8]. Controls were approached by researchers at the airport at check-in using the same method as for cases. All participants completed the same anonymous questionnaire including basic demographics, sexual behaviour prior to and during their stay in Ibiza (whether visiting with a sexual partner, number of sexual partners, use of condoms, whether they had sex with a non-UK resident) and alcohol and drug use (frequency of use of cannabis, ecstasy, cocaine, amphetamine, ketamine, gammahydroxybutyrate, LSD, opiates[8,9]) in Ibiza and the UK. Controls who reported visiting Ibiza for work purposes or staying for a period greater than 90 days were excluded from analyses (n = 40). All analyses were undertaken using SPSS and utilised *X*^2^, Mann Whitney U tests and logistic regression to compare sexual behaviour within cases and between cases and holidaymaking controls for demographics and factors associated with increased likelihood of sex, unprotected sex and higher numbers of sexual partners. Having unprotected sex in Ibiza is defined as not always using a condom when having sex with any person on the island.

## Results

There were no significant differences between cases and controls for gender (cases 55.4%, controls 60.7% male, *X*^2 ^= 0.949, *P *= 0.330) or median age (cases 21 (range 18–32) controls 21 (range 16–35), *Z *= 0.356, *P *= 0.722). However median length of stay in Ibiza was significantly longer for cases (cases 100 days, controls 7 days, *Z *= 16.845, *P *< 0.001). Cases were more likely than controls to have used illegal drugs in both UK and Ibiza, however no differences were found in alcohol consumption with very high levels reported by both groups in Ibiza and UK (Table 1).

Basic chi squared analyses of sexual behaviour found significant differences between the sexual behaviour of cases and controls. Overall cases were more likely to report having sex in Ibiza. Comparing only those that arrived without a sexual partner, cases were still more likely to have sex in Ibiza, have sex with a non-UK resident and have sex with a greater number of people in Ibiza and in the six months before visiting the island (Table 1). Limiting the analysis further to those that arrived without a sexual partner and had sex in Ibiza, cases were more likely to have had unprotected sex at all and to have had unprotected sex with more people. However, they had fewer sexual partners per week of stay in Ibiza than holidaymakers.

For those arriving without a sexual partner, logistic regression was used to control for potential confounding differences between cases and controls (age, sex, drug use in Ibiza and number of sexual partners in the six months prior to visiting the island; Table 2). Results confirmed that being a casual worker was independently associated with having sex in Ibiza, having unprotected sex and having multiple sexual partners. However, there was no significant difference between casual workers and holidaymakers in having sex with a non-UK resident. Having sex and having multiple sexual partners in Ibiza were also strongly related to the number of sexual partners individuals had in the previous six months (Table 2).

To explore the relationship between drug use and sexual behaviour of casual workers further, sexual behaviour was analysed by individual drugs of use in Ibiza (cannabis, ecstasy, cocaine, amphetamine, LSD, ketamine, gammahydroxybutyrate, opiates). For those arriving without a sexual partner, there was no relationship between any type of drug used and having had sex. However, of those that had sex in Ibiza, cases who used amphetamine during their stay were more likely to have had multiple sexual partners (>1 partner, 91.7% amphetamine users, 58.8% non-users, X^2 ^= 7.597, P < 0.01). There were no differences in levels of unprotected sex among cases that related to individual drug types used. The relationship between drugs used and sexual behaviour of holidaymakers in Ibiza have been discussed elsewhere[8].

## Discussion

Increasing availability of cheap international travel means more young people are spending time abroad, with foreign resorts associated with nightlife among the most popular destinations[10]. In turn this creates greater opportunity for young people to extend visits abroad with casual work such as bar service or nightclub promotion. These individuals can be central to setting social norms in holiday resorts. They are often among the first individuals holidaymakers encounter on arrival at a resort and can be a source of advice and information for peers. However, their extended stay means they are exposed to the hedonistic lifestyle of international nightlife resorts for long periods of time. Despite this, little is known about their sexual behaviour while abroad.

Here we have analysed the sexual behaviour of this important group and compared it to that of those travelling simply for holidaymaking purposes. As with all questionnaire based studies on sexual behaviour, the findings reported here may be affected by compliance rates and over or under reporting of sexual activity, often through social pressures to exaggerate or understate sexual activity[11]. To minimise such confounding effects, we employed a standardised methodology that involved explaining the survey objectives to potential respondents, providing assurances of confidentiality and allowing individuals to complete and return questionnaires through a mechanism that clearly maintained their anonymity. Cases were also biased toward those working in San Antonio, a town particularly noted for its association with clubbing and nightlife. While such individuals may not be typical of all tourism or even nightlife resorts, they do represent those working in a key tourist location for UK youth.

Our results show that both young British people working in bars and nightclubs in Ibiza and those visiting solely for holiday purposes display high levels of sexual risk taking while abroad (Table 1). Compared with holidaymakers, however, casual workers are more likely to have sex, to have unprotected sex and to have greater numbers of sexual partners during their stay (Table 2). Four in five have sex with one or more new partners in Ibiza, and two thirds of these have unprotected sex. Over a quarter have sex with a non-UK resident. Longer periods spent abroad provide casual workers with greater opportunity for meeting new partners. However, these individuals have fewer partners per week than holidaymakers, consistent with findings of elevated sexual behaviour in the first few weeks of employment in domestic holiday resorts before casual workers settle in to their new environments[12]. Thus, this initial period of employment may be an essential time for delivering interventions to young people at high risk of sexually transmitted infections and unwanted pregnancy.

Casual workers are also ideally placed to deliver health interventions to other young travellers. Although fewer holidaymakers reported sexual risk taking in Ibiza, levels of both sexual activity and unprotected sex were high and were concentrated in a much shorter period. In an average seven-day stay, two in every five holidaymakers who arrived without a sexual partner had sex and of these 40% had unprotected sex. Thus this group also constitutes an essential target for sexual health promotion[8]. Casual workers can have high status in holiday resorts, greater knowledge of local services and cultures, and firsthand experience of holiday behaviour. Their presence throughout the summer season means they should be considered key partners in sexual health promotion and interventions such as peer education and delivery of sexual health information materials and condoms.

Any intervention to address sexual risk taking among young people abroad needs to take into account the high levels of alcohol and drug use during holiday periods. Here few associations were found between substance use and sexual behaviour. However, the near ubiquitous consumption of alcohol in particular means that it is difficult to measure its effect on sexual behaviour and safe sex and elsewhere alcohol has been linked to unprotected and regretted sex[13,14]. Sexual health information should highlight the increased risks to sexual health associated with substance use and be targeted in locations where alcohol, drug use and consequent casual sexual relationships occur.

Most nightlife and peer led interventions to promote sexual health target at risk populations while at home. Interventions abroad are complicated by the expense of transporting and accommodating peer or other workers and the sheer quantity of individuals travelling abroad and participating in nightlife on a daily basis. Consequently delivering sexual health interventions abroad will require close collaboration with public health and sexual health organisations in other countries in order to identify where local services can be supported to meet the needs of tourists. Further, where peer interventions are delivered, a limited capacity means focusing on those most at risk and those most likely to act as social mediators for the wider dance music tourism population. This work shows casual workers are a key group. However more research is required on which interventions will prove most effective at changing their behaviour and empowering them to change the behaviour of others.

## Conclusion

Sexually transmitted infections are continuing to increase in many countries, particularly among young people, and new approaches to tackling risky sexual behaviour are urgently needed. Although relationships between travel and sexual health problems are currently difficult to quantify, research consistently links travel, sexual behaviour and sexually transmitted infections[15]. Trends in recreation and travel mean millions of young people now spend time abroad where they have greater opportunity to meet new sexual partners in atmospheres of widespread substance use and reduced social responsibilities. The sexual health needs of young people abroad cannot be ignored, particularly when those at most risk of sexual health problems play such central roles in holiday resorts. Casual workers in bars and nightclubs in international nightlife resorts not only display higher levels of sexual risk taking abroad, but also have higher numbers of sexual partners at home (see Table 1), making them potential conduits for the international spread of sexually transmitted infections. Targeting these individuals as both recipients and providers of sexual health interventions while abroad will provide easier access to those young people most at risk of sexual health at a time when health information may be most pertinent.

## Competing interests

The author(s) declare that they have no competing interests.

## Authors' contributions

KH participated in the design of the study and data collection, analysed the data and wrote the manuscript. MAB designed the study and participated in data analysis and writing the manuscript.

## Pre-publication history

The pre-publication history for this paper can be accessed here:



## Figures and Tables

**Table 1:** Sexual behaviour in Ibiza and UK by casual workers in Ibiza (cases) and holidaymakers (controls)

		**Cases**		**Controls**			
		%	n	%	n	*X*^2^	P
Total sample		92		828			
Visited Ibiza with partner (%)	Yes	15.4	91	23.2	793	2.871	0.090
Had sex in Ibiza (%)	Yes	81.5	92	49.7	729	33.317	<0.001
Used alcohol in:							
Ibiza (%)	Yes	98.9	92	98.4	819	0.137	0.711
UK (%)	Yes	97.8	89	99.0	822	1.201	0.273
Used drugs in:							
Ibiza (%)	Yes	95.7	92	51.2	819	66.339	<0.001
UK (%)	Yes	95.6	90	57.4	820	49.624	<0.001

***Those arriving in Ibiza without a partner only***							
Sub sample		77		610			
Had sex in Ibiza (%)	Yes	80.5	77	40.7	550	43.112	<0.001
Number of sexual partners (%)	0	20.5	73	59.4	549		
	1	21.9		19.1			
	2–5	34.2		13.7			
	6+	23.3		7.8		50.919	<0.001
Had sex with non UK resident in Ibiza (%)*	Yes	27.4	73	11.4	500	14.014	<0.001
Number of sexual partners in 6 months prior to visiting Ibiza (%)	0	2.8	72	14.3	546		
	1	26.4		39.4			
	2–5	56.9		36.8			
	6+	13.9		9.5		17.133	<0.001

***Those arriving in Ibiza without a sexual partner and having sex in Ibiza only***							
Sub sample		62		224			
Had unprotected sex	Yes	65.5	58	40.4	223	11.746	<0.005
Number of unprotected partners in Ibiza(%)	0	34.5	58	59.6	223		
	1	17.2		20.2			
	2–5	34.5		12.1			
	6+	13.8		8.0		20.959	<0.001
Number of sexual partners per week spent in Ibiza (%)	<1	91.1	56	19.8	222		
	1–2	5.4		50.0			
	>2	3.6		30.2		100.927	<0.001

n = number of respondents

* Respondents providing a "don't know" response were classified as no for this analysis (n = 28)

**Table 2:** Logistic regression for sexual behaviour in Ibiza among individuals arriving without a sexual partner

	**Had sex**			Had unprotected sex*			Had >1 partner*			Had sex with a non-UK resident*		
	AOR	95%CI	P	AOR	95%CI	P	AOR	95%CI	P	AOR	95%CI	P
**Sample type**												
*Holidaymaker*	ref			ref			ref			ref		
*Casual worker*	5.23	2.78–9.84	<0.001	3.01	1.61–5.63	<0.005	2.82	1.41–5.65	<0.005	NS		
**Gender**												
*Female*	ref			ref			ref			ref		
*Male*	NS			NS			2.19	1.23–2.90	<0.01	2.4	1.20–4.77	<0.05
**Age group**												
*20+*	ref			ref			ref			ref		
*16–19*	1.89	1.27–2.80	<0.005	NS			NS			NS		
**Sexual partners in previous 6 months**												
*0*	ref		<0.001	ref			ref		<0.001	ref		<0.005
*1*	1.47	0.80–2.73	NS	NS			6.21	1.29–30.04	<0.05	0.84	0.23–3.01	NS
*2–5*	3.91	2.12–7.19	<0.001	NS			7.19	1.54–33.51	<0.05	0.73	0.22–2.48	NS
*6+*	21.29	8.38–54.09	<0.001	NS			46.86	8.34–263.19	<0.001	2.72	1.20–4.77	NS
**Used drugs in Ibiza**												
*No*	ref			ref			ref			ref		
*Yes*	NS			NS			NS			NS		

NS = not significant. AOR = adjusted odds ratios. CI = confidence intervals. Reference categories for each variable are identified with (ref). P values placed next to the reference category for Sexual partners in the previous 6 months refer to the overall significance across categories.

*Analyses of having had unprotected sex, sex with more than one partner and sex with a non-UK resident in Ibiza are limited to individuals who arrived without a sexual partner and had sex while abroad.
